# Application of Support Vector Machine on fMRI Data as Biomarkers in Schizophrenia Diagnosis: A Systematic Review

**DOI:** 10.3389/fpsyt.2020.00588

**Published:** 2020-06-23

**Authors:** Luca Steardo, Elvira Anna Carbone, Renato de Filippis, Claudia Pisanu, Cristina Segura-Garcia, Alessio Squassina, Pasquale De Fazio, Luca Steardo

**Affiliations:** ^1^Department of Health Sciences, School of Medicine and Surgery, University Magna Graecia of Catanzaro, Catanzaro, Italy; ^2^Section of Neuroscience and Clinical Pharmacology, Department of Biomedical Sciences, Faculty of Medicine and Surgery, University of Cagliari, Cagliari, Italy; ^3^Department of Medical and Surgical Science, University of Magna Graecia, Catanzaro, Italy; ^4^Department of Psychiatry, Faculty of Medicine, Dalhousie University, Halifax, NS, Canada; ^5^Department of Physiology and Pharmacology, Faculty of Pharmacy and Medicine, Sapienza University of Rome, Rome, Italy; ^6^Department of Psychiatry, Giustino Fortunato University, Benevento, Italy

**Keywords:** machine learning, schizophrenia, support vector machine (SVM), resting-state fMRI, biomarkers

## Abstract

Non-invasive measurements of brain function and structure as neuroimaging in patients with mental illnesses are useful and powerful tools for studying discriminatory biomarkers. To date, functional MRI (fMRI), structural MRI (sMRI) represent the most used techniques to provide multiple perspectives on brain function, structure, and their connectivity. Recently, there has been rising attention in using machine‐learning (ML) techniques, pattern recognition methods, applied to neuroimaging data to characterize disease-related alterations in brain structure and function and to identify phenotypes, for example, for translation into clinical and early diagnosis. Our aim was to provide a systematic review according to the PRISMA statement of Support Vector Machine (SVM) techniques in making diagnostic discrimination between SCZ patients from healthy controls using neuroimaging data from functional MRI as input. We included studies using SVM as ML techniques with patients diagnosed with Schizophrenia. From an initial sample of 660 papers, at the end of the screening process, 22 articles were selected, and included in our review. This technique can be a valid, inexpensive, and non-invasive support to recognize and detect patients at an early stage, compared to any currently available assessment or clinical diagnostic methods in order to save crucial time. The higher accuracy of SVM models and the new integrated methods of ML techniques could play a decisive role to detect patients with SCZ or other major psychiatric disorders in the early stages of the disease or to potentially determine their neuroimaging risk factors in the near future.

## Introduction

Schizophrenia (SCZ) is a major psychiatric disorder characterized by positive and negative symptoms, associated with cognitive impairment, leading to a worse outcome and a high impact on global functioning (1). The lifetime prevalence is 0.40% (2), and it has been estimated that approximately 1 in 200 individuals will be diagnosed with SCZ at some point during their lifetime (3). Even if the diagnosis of schizophrenia is made by observation of the clinical features of the disorder according to the Diagnostic and Statistical Manual of Mental Disorders 5 (DSM-5) (4) or on the ICD (5) criteria, evidences on specific biomarkers that can predict or detect the disease accurately at an early stage are still scarce. (6). It is clear that, considering the biological complexity, the attempt to improve insights into the disease processes is difficult: brain neuroanatomy is intrinsically complex and heterogeneous (7). Non-invasive measurements of brain function and structure, as neuroimaging, are useful and powerful tools for studying discriminatory biomarkers (8, 9) in patients with mental disorders. In this regard, brain imaging studies have revealed that functional and structural brain connectivity in the default mode network (DMN), salience network (SN) and central executive network (CEN) are consistently altered in schizophrenia (10). To date, functional MRI (fMRI) and structural MRI (sMRI) represent the most used techniques to provide a multiple perspective on brain function, structure, and its connectivity. Large amounts of imaging data from magnetic resonance imaging (MRI) need to be analyzed by computerized methods that are able to process information and determine the probability of diseases with great precision (11). Rising attention has been given to machine‐learning (ML) techniques (i.e. pattern recognition methods) applied to neuroimaging data (12) to identify phenotypes to be translated into clinical practice for early diagnosis (13, 14). ML techniques applied to fMRI analyze highly complex data sets and assess the importance and interactions between variables, exploring brain functionality and making accurate predictions (15, 16). Machine learning stems from the theory that computers can learn to perform specific tasks without being programmed to do so starting from specific input, thanks to the recognition of patterns in the data. Machine learning uses algorithms that learn from data iteratively. For example, it allows computers to find information, even unknown, without being explicitly told where to look for it (17). Among them, the Support Vector Machine (SVM) represents one of the ML techniques that has shown higher accuracy and precision especially in predicting clinical outcome and severity in schizophrenia patients (14). SVM is a supervised learning model with associated learning algorithms that analyzes data used for classification and regression analysis. This technique has yielded good results applied to fMRI in defining a set of features and information from the various regions of the brain allowing to classify healthy controls and patients affected by SCZ with a potential great translational impact (11).

This review aimed to assess the current state of the evidence about the use of SVM techniques in making diagnostic discrimination in SCZ patients from healthy controls (HC) using as input neuroimaging data from fMRI, according to PRISMA guidelines (18).

## Materials and Methods

### Search Strategy

Articles published until September 27^th^, 2019 in PubMed, Embase, MEDLINE, PsychINFO, and the Cochrane Library, without language and time limits, were searched by using the following keywords: (*Deep Learning OR DL OR Big data OR Artificial Intelligence OR Machine Learning OR Gaussian process OR Regularized logistic OR Linear discriminant analysis OR LDA OR Random forest OR Least Absolute selection shrinkage operator OR elastic net OR LASSO OR RVM OR relevance vector machine OR pattern recognition OR Computational Intelligence OR Machine Intelligence OR support vector OR SVM OR Pattern classification OR Deep learning*) AND *Schizophrenia* AND (*fMRI OR magnetic resonance imaging OR MRI OR functional MRI OR functional-MRI OR functional magnetic resonance imaging*). All the selected studies were individually reviewed by two researchers. Reference lists from the included articles were screened for additional studies. The eligible publications have been included and cited in this review.

### Assessment of Study Quality

In this systematic review we applied the Jadad rating system (19) to check the methodological quality of included studies. Jadad's process allows to qualify selected studies according to their transparency and reproducibility, with great validity and reliability evidence, through the description of three simple and easy items: randomization methods, the double-blinding procedure, and the patient's withdrawal and dropout reports. Scores range from 0 to 5 points. The cut-off for inclusion in this study was a Jadad score ≥3.

### Selection Criteria

We selected studies applying SVM as ML techniques with patients diagnosed with Schizophrenia according to the DSM-IV, DSM-IV TR, DSM-5 or ICD-10 criteria, chronic SCZ or at first episode of schizophrenia (FES) regardless of antipsychotic medications. We excluded studies without a control group and trials including patients affected by general medical conditions, neurological or psychiatric comorbidity, substance abuse or alcohol dependence, traumatic brain injuries with loss of consciousness, and unclear or unverified psychiatric diagnoses according to the DSM or ICD criteria.

### Data Collection and Extraction

Two authors (RdF and EAC) independently screened all the titles and abstracts of the collected articles, and fully read the texts of papers that met the eligibility criteria. In cases of disagreement, a third researcher (LS) supervised and made the final decision. Data from the extracted article included: publication year, sample size, diagnoses, and all statistical data and features (i.e. accuracy, sensitivity, specificity, brain region or networks).

## Results

**Figure 1:**
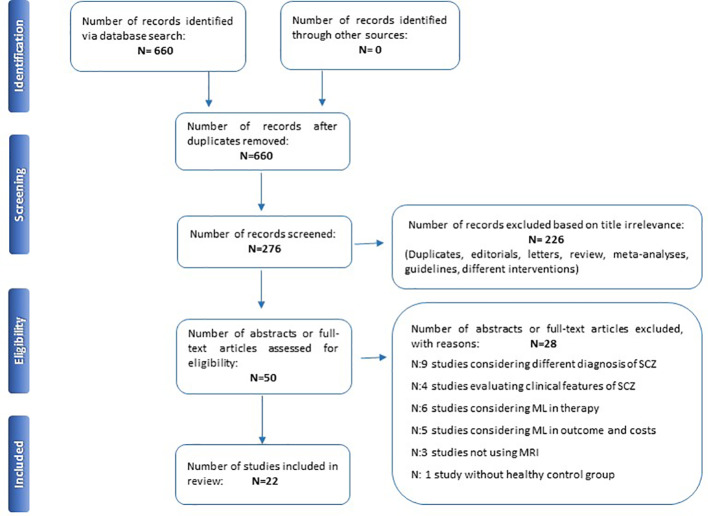
PRISMA flowchart of included studies.

Initially, 660 items were identified, of which 384 articles were eliminated because they did not fulfill the inclusion criteria. The abstracts of the remaining 276 articles were reviewed. Overall, 226 out of 276 articles were excluded because they were not trials (i.e. editorials, letters to editors, reviews, meta-analyses, case reports or different interventions). Then, 28 manuscripts out of 50 papers were further excluded because they did not fulfill the inclusion criteria (e.g. unclear or unverified psychiatric diagnoses, studies considering outcome, costs or therapy or not using MRI); the remaining 22 studies (**Table 1**) were included in this review (**Figure 1**).

**Table 1:** Summary of included studies classifying schizophrenia using SVM.

Author, year	Sample size	Best accuracy	Other measures (sensibility, speciﬁcity, AUC)	Data features as input	Brain regions and networks involved	Jadad's score	Comments
**Su et al**. (20)	N: 64 – 32 SCZ – 32 HC	82,8%	Sp: 81, 2% Sp :84, 4%	90 regions (45 for each hemisphere) and 26 areas (nine in each cerebellar hemisphere and eight in the vermis)	Default mode network, cerebellum, visual network, sensorimotor network, fronto-parietal network, cingulo-opercular network	4	The trial is conﬁned to connectivity analyses.
**Yang et al**. (21)	N: 40 – 20 SCZ – 20 HC	Hybrid ML 87,3%	Sn: 85,8% Sp: 88.8%	150 SNPs from a database + auditory stimuli	Cingulate gyrus, post-/pre-central gyrus, para-central lobule, precuneus, superior and inferior parietal lobule.	5	Hybrid ML technique using together fMRI and SNP data for more accuracy.
**Arbabshirani et al**. (22)	N: 370 – 195 SCZ – 175 HC	88,2%	Sn: 86,7% Sp: 89,5%	1128 features for each subject extracted	Control processes, default-mode, cerebellar networks, and subcortical, auditory, visual, somatomotor regions.	5	Functional network connectivity and autoconnectivity improved signiﬁcantly classiﬁcation results.
**Watanabe et al**. (23)	N: 91 – 54 SCZ – 67 HC	77–88.2%	N.A.	Authors described a whole brain resting state functional connectome.	Lateral prefrontal cortex, intra-frontoparietal, frontoparietal default, intracerebellum networks.	3	Authors assessed three sets of network-to-network connections as their role in SZ psychopathology was considered crucial.
**Koch et al**. (24)	N: 98 – 44 SCZ – 54 HC	69.3–93.2%	Sn: 70.5–100% Sp: 40.9–93.2%	Six fMRI volumes per trial were acquired, resulting in a total of 450 volumes per run	Nucleus accumbens, amygdala, insula, thalamus, ventral striatum, right pallidum, putamen, right inferior frontal gyrus, inferior temporal gyrus	5	Able to use the ventricular striatal activation patterns to predict the severity of the negative symptoms of patients enrolled.
**Chyzhyk et al**. (25)	N: 147 – 72 SCZ – 75 HC	≈90%	N.A.	rs-fMRI data were collected with single-shot full k-space EPI with ramp sampling correction using the AC-PC as a reference.	Inferior temporal gyrus, para-hippocampal gyrus, planum polare, thalamus, temporal fusiform cortex	4	Application of SVM and RF methods to the data extracted from cross-validation as the ensembles of ELM
**Liu et al**. (26)	N: 79 – 48 Drug-naïve SCZ – 31 HC	89.9%	Sn: 91.67% Sp: 87.10%	The fMRI scan lasted for 480 s for every pt included, and in total 240 volumes were obtained. The ﬁrst 10 volumes of each take-over were discarded to certain steady state conditions	Left paracentral lobule, left postcentral gyrus, left superior temporal gyrus, right middle frontal gyrus, bilateral precuneus, right pre-central gyrus, right inferior parietal lobule.	4	Authors enrolled only adolescent onset without previous medication SCZ patients
**Guo et al**. (27)	N: 96 – 28 SCZ – 40 HC – 28 relatives	94.6%	Sn: 92.9% Sp: 96.4%	Long-range and short-range FCs	Default-mode network, left fusiform gyrus, cerebellum, sensorimotor circuits, right superior parietal lobule	4	The SCZ group was unmedicated and recent onset, so, results may be confounded by their acute positive symptoms
**Orban et al**. (28)	N: 382 – 191 SCZ – 191 HC	84%	N.A.	Functional brain connectomes included a total of 2016 functional connections among 64 brain parcels	Connectivity of the whole brain	3	Brain imaging data derived from six different and independent studies and databases.
**Wang et al**. (29)	N: 79 – 48 AOS – 31 HC	90.1%	Sn: 88.2% Sp: 91.9%	Authors used brain regions with signiﬁcantly different ReHo values between SCZ and HC group	Bilateral superior medial pre-frontal cortex, right inferior parietal lobule, left paracentral lobule, left superior temporal gyrus, right precentral lobule	5	Sample sizes in the two groups were different.
**Wang et al**. (30)	N: 79 – 48 drug-naïve – 31 HC	92.4%	Sn: 89.6% Sp: 96.8%	Regional homogeneity (ReHo), a measurement that reﬂects brain local functional connectivity or synchronization	Left superior temporal gyrus, right middle frontal gyrus, right superior medial prefrontal cortex	4	SVM analysis was applied to an independent database
**Bae et al**. (31)	N: 75 – 21 SCZ – 54 HC	92.1%	Sn: 92.0% Sp: 92.1% Precision: 94%	90 ROIs from the image database.	Anterior right cingulate cortex, inferior left parietal region, superior right temporal region	5	Likely interference of pharmacological treatment and disease phase on the investigated functional connections. Moreover, authors used only n-back tests without rs-fMRI
**Qureshi et al**. (32)	N: 144 – 72 SCZ – 72 HC	99.3%	Sn: 100% Sp: 98, 6%	Mean cortical thickness, white matter volume, surface area, volume, cortical thickness standard deviation, mean curvature, subcortical segment volume, subcortical intensity, and overall brain volume and intensity as the structural features	Surface area, cortical thickness, global average functional connectivity , WM/subcortical/overall volume, curvature	4	Authors developed a specific ELM in this trial
**Pläschke et al**. (33)	N: 170 – 86 SCZ – 84 HC	61–72%	Sn: 65–77% Sp: 46–69% AUC: 0.61–0.79	12 functional networks.	Emotion-processing, empathy, and cognitive action control networks	3	Young-old classiﬁcation was grounded on outperformed clinical classiﬁcation and all networks.
**Liu et al**. (34)	N: 79 − 48 Drug-Naïve FES – 31 HC	94.93%	Sn: 100% Sp: 87.09%	A total of 240 volumes were acquired. The ﬁrst 10 volumes of each scan were discarded to certain steady-state conditions at the beginning of acquisition.	Superior temporal gyrus, insula, fusiform gyrus, precentral gyrus, and precuneus	4	Authors assessed also a neurocognitive test battery demonstrating neurocognitive deficits in patients compared to HC
**Vacca et al**. (35)	N: 201 – 86 SCZ – 115 HC	87,8%	N.A.	Battery tests related to attention, memory, praxic, visuospatial and executive functions	Working memory, executive functions, attention, verbal fluency, memory	3	Data obtained should be integrated thorough neuropsychological evaluation into the more general diagnostic approach of patients with SCZ
**Zhuang et al**. (36)	N: 69 – 40 drug naïve FES – 29 HC	84,29%	Sn:92.5 Sp: 73.33	Structural MRI , diffusion tensor imaging (DTI) and rs-fMRI data	Altered morphological measurements in both gray matter and white matter, functional connectivity, and regional functional activity	5	A multimodal classification method to discriminate FES schizophrenia patients from HC by a combined structural MRI, DTI, and rs-fMRI data
**Li et al**. (37)	N: 148 – 60 SCZ – 71 HC	71,8%	Sn: 70 Sp: 73,24	Aberrant connectivities in both intra- and inter-hemispherical connections	Disconnectivities mainly appeared on temporal and occipital regions for the within-large-region connections; connectivity disruption was observed on the connections from temporal region to occipital, insula and limbic regions for the between-large-region connections	4	The findings of this study corroborate previous conclusion of dysconnectivity in SCZ and further shed light on distribution patterns of dysconnectivity, which deepens the understanding of its pathological mechanism.
**Jing et al**. (38)	N: 153 – 60 SCZ – 43 unaffected FDRs of patients – 50 HC	83,9%	Sn: 87.5% Sp: 80.0% AUC: 0.914	Informative FNs	Cerebellum, default mode network (DMN), ventral frontotemporal network, and posterior DMN with parahippocampal gyrus	5	Pattern classifiers built upon the informative FNs can serve as biomarkers for quantifying brain alterations in SCZ and help to identify FDRs with FN patterns and cognitive impairment similar to those of SCZ patients.
**Ramkiran et al**. (39)	N: 112 – 56 SCZ – 56 HC	69%	Sn: 68% Sp: 72%	Functional connectivity	The basal ganglia, thalamus, lingual gyrus, and cerebellar vermis showed signiﬁcantly diﬀerent, type A (decreased anticorrelation) connections. The medial temporal lobe and posterior cingulate gyri showed signiﬁcantly diﬀerent, Type B (increased anticorrelation) connections.	4	Different aberrant functional connectivity in SCZ patients.
**Ji et al**. (40)	N: 737 – 240 HC – 161 Bipolar – 131 schizoaffective – 205 SCZ	64%	AUC: 69%	Whole brain ReHo	Inferior/middle temporal area and fusiform gyrus	5	Patterns of higher ReHo abnormalities could be used as robust psychosis biomarker.
**Zhu et al**. (41)	N: 221 – 76 FES drug naïve – 74 ultra-high risk – 71 HC	74,83%	Sn: 68, 42% Sp: 81, 69%	Parameter of functional asymmetry	Left thalamus/pallidum, right hippocampus/parahippocampus, right inferior frontal gyrus/insula, right thalamus, and left inferior parietal lobule-right precentral gyrus/postcentral gyrus, and left calcarine, right superior occipital gyrus/middle occipital gyrus.	3	First-episode patients and UHR subjects shared decreased PAS in the left thalamus. This observed pattern of functional asymmetry highlights the involvement of the thalamus in the pathophysiology of psychosis and could be also applied as a very early marker for psychosis.

AOS., adolescent onset SCZ; AUC, area under the curve; DMN, default mode network; DTI, diffusion tensor imaging; ELM, extreme learning machine; FC, functional connectivity; FN, functional network; FDR, first-degree relatives; FES, first episode schizophrenia; HC, healthy controls; ML, machine learning; n, number; NA, not available; PAS, parameter of asymmetry; RF, random forest; ReHo, regional homogeneity; ROI, region of interest; SCZ, schizophrenia; SN, sensibility; SNP, single nucleotide polymorphisms; SP, specificity; SVM, support vector machine; UHR, ultra-high risk.

## Discussion

Included studies were very heterogeneous, and the samples vary in size and clinical characteristics (**Table 1**). Several features from different brain regions were used as inputs for SVM and focused to investigate how the performance of the model in accuracy, precision, sensitivity, and specificity could be affected by these variables. Studies in this review mostly used and evaluated frontal, temporal, and occipital brain regions. ML techniques were able to detect signiﬁcantly altered activation patterns or brain connectivity differences in SCZ patients compared to HC. Moreover, this happened quickly, effectively, and efficiently, greatly reducing the number of false negatives, as desirable for a good screening test (42, 43). SVM has achieved good results in terms of accuracy and precision in identifying patients with SCZ. This technique can improve the clinical and research tasks due to the repetitiveness of the data. Computers learn from previous processing to produce results and make decisions that are reliable and replicable (17). SVM presents pros and cons. Specifically, an important advantage is that SVM is the most used and well-known machine learning tool, and even when other techniques are validated, they are compared with SVM. It achieves high accuracy level (e.g. 99%) and is the golden standard to develop new techniques. It can be used for both classification and regression purposes; it allows data repeatability; it can be used in different fields of study, and it represents a great option for future studies. However, it is expensive, and its interpretation is not simple as it requires an experienced and dedicated team (14, 44, 45).

Pläschke et al. used the resting-state Functional Connectivity (FC) to differentiate SCZ patients from matched HC, reaching a remarkable accuracy, equal to 68%. Interestingly, emotional scenes and face processing, empathic processing, and cognitive action control have proven to be the best networks to accurately discriminate patients from HC. Moreover, the age affects network integrity in a more global way so it could be used as a specific flag of functional dysregulation in particular networks affected in SCZ (33). The results of Bae's study reported a decrease in the global and local network connectivity in SCZ patients compared with HC, especially in the superior right temporal region, in the anterior right cingulate cortex, and the inferior left parietal region with an accuracy of 92.1%, sensitivity of 92%, speciﬁcity of 92.1% and precision 94% (31). One of the largest studies on SCZ (200 patients vs 200 HC) reported a high diagnostic accuracy (84%) using data from several locations. Otherwise, signiﬁcantly poorer accuracy was reached with the use of individual sites, showing a lower connectivity in SCZ patients (28). Su et al. recreated the whole brain functional connectivity in SCZ patients (23) vs HC (23) and related the exact spatial location of the activated brain areas to the emerging symptoms. With >80% accuracy authors found an increased FC in SCZ patients group (20). It could probably be explained by an altered cerebral connectivity spread throughout the whole brain, with particular aberrations found in many of the main connections. Altered connectivities in both intra- and inter-hemispherical connections were observed by Li et al. (37), especially in the right hemisphere more than the left hemisphere (temporal, occipital, insula, and limbic regions). Similar data were confirmed in others studies focusing on altered connections (decreased in the basal ganglia, thalamus, lingual gyrus, and cerebellar vermis and increased in medial temporal lobe and posterior cingulate gyri) (39). Koch et al. reached 93% accuracy in identifying SCZ patients and were also able to predict the severity of the negative symptoms of patients based on ventricular striatal activation patterns (24). The results of these studies corroborate the idea of the occurrence of dysconnectivity in schizophrenic patients and deepen our knowledge on the pathological mechanisms.

Functional network connectivity (FNC) to capture the internetwork connectivity pattern and autoconnectivity to capture the temporal connectivity of each brain network were proposed as features for SVM technique (22). The authors manage to achieve particularly high accuracy values in order to discriminate patients with SCZ from HC thanks to the integration of these features (autoconnectivity + FNC). Indeed, the final diagnostic and classiﬁcation accuracy settles in 88.21% (83.7% for FNC and 80.2% for autoconnectivity alone), with a sensitivity of 86.7% (81.4% for FNC and 78.1% for autoconnectivity alone) and a speciﬁcity of 89.5% (85.9% for FNC and 82.2% for autoconnectivity alone). In one of the first studies, the authors were able to analyze the whole functional connectome both in the patient and in the HC groups. They demonstrated many of the main differences, although general and poorly detailed. Indeed, they weighed three series of network-to-network connections (intra-frontoparietal, intra-cerebellar, frontoparietal default) considered to be of major importance for SCZ psychopathology and clinical manifestation (23). Another paper examined the role of long- and short range functional connectivity (lFC) (sFC) in discriminating patients from their own relatives or HC: SCZ group exhibited an spread in sFC and lFC in the DMN with an adequate level of accuracy, sensitivity, and speciﬁcity (94%, 92%, 96%, respectively) (27). By analyzing the coherence regional homogeneity (Cohe-ReHo) value, Liu et al. demonstrated that it was decreased in several areas, such as the left postcentral gyrus, right precentral gyrus, left superior temporal gyrus, right middle frontal gyrus, left paracentral lobule, right IPL, and bilateral praecuneus in 48 SCZ vs 31 HC (26). The Whole brain ReHo measures were used as robust psychosis biomarker: SVM resulted more accurate in identify patterns of higher ReHo abnormalities (inferior/middle temporal area and fusiform gyrus) (40). The integration of the neuropsychological evaluation to detect different aspects related to attention, working memory, praxic, visuospatial, and executive functions was able for the early diagnosis of patients with SCZ (35).

The combination of SVM with other ML techniques can identify anatomic brain areas with major alterations (temporal fusiform cortex, inferior, middle, and medial frontal gyri, inferior temporal gyrus, anterior division of the parahippocampal gyrus, planum polare, cingulate gyrus, superior temporal gyrus, precuneus left, and right thalamus) with an accuracy close to 90% (21, 25). An extreme learning machine (ELM) was developed by Qureshi et colleagues, reaching a maximum accuracy of 99.3%. Main data derived from cortical thickness and surface area, total cerebral volume, and overall volume of cortex features scans. Authors concluded that their ELM technique can be applied to patients offering a solid chance of helping clinicians to make diagnosis of SCZ (32).

Another important field of application of SVM is the evaluation of functional features in first episode schizophrenia (FES). The identification of early-onset schizophrenia remains challenging, and SVM may constitute a promising tool for the early diagnosis for its high accuracy and valuable prognostic implication in FES. Recently, the sFC and lFC in the whole brain were explored in 48 ﬁrst-episode, drug-naïve patients and 31 HC using SVM. Major abnormalities were found in some brain networks (anterior and posterior Default Mode Network and Sensorimotor Network) classifying patients and controls with > 92% accuracy and high sensitivity and speciﬁcity (30). Liu et al. evaluated the alteration in FC in different brain regions in a similar patients' sample and found dysfunctional interhemispheric network within the sensorimotor area among patients with SCZ. It was associated with processing speed deﬁcits, indicating the probable involvement with the neurocognitive alterations of these patients. The application of SVM ML technique analysis reached 100% sensitivity, 87.09% speciﬁcity, and 94.93% accuracy (34). Functional alterations could point to a role of DMN and SN in the SCZ psychopathology that is already known in first-psychotic episode patients and SVM seems to be able to discriminate with high accuracy patients from HC in research context. Wang et al. identify brain peculiarities using ReHo input in SVM analysis through resting state-fMRI (rs-fMRI) in drug-naïve patients and 32 HC. ReHo values were signiﬁcantly amplified in the bilateral superior medial prefrontal cortex, and, otherwise, reduced in the left superior temporal gyrus, right precentral lobule, right inferior parietal lobule, and left paracentral lobule in patient group compared to HC (29). Disrupted functional asymmetry was calculated comparing patients with FES, drug-naïve schizophrenia, ultra-high risk (UHR) for psychosis and HC. SVM classiﬁcation analysis was applied to analyze the data and showed decreased parameter of asymmetry in the left thalamus/pallidum, right hippocampus/parahippocampus, right inferior frontal gyrus/insula, right thalamus, and left inferior parietal lobule, and increased PAS in the left calcarine, right superior occipital gyrus/middle occipital gyrus, and right precentral gyrus/postcentral gyrus. First-episode patients and UHR subjects shared decreased pattern of functional asymmetry in the left thalamus underlining the possible involvement of the thalamus in the pathophysiology of psychosis and demonstrating a very early marker for psychosis (41). A multimodal classification method to discriminate FES patients from HC combined structural MRI and rs-fMRI data, and identified functional markers in both gray matter and white matter and altered functional connectivity in DMN and cerebellar connections (36). A recent study identified informative functional networks to distinguish patients from HC and to classify unaffected first-degree relatives (FDRs) with or without functional networks similar to patients. Four informative functional networks (DMN, ventral frontotemporal network, and posterior DMN with parahippocampal gyrus) resulted implicated in brain alterations. They could be probably used as biomarkers to identify FDRs with FN patterns similar to those of SCZ patients (38). The ability to apply complex mathematical calculations to big data is newly developed, and its use is hopefully growing. Now, theoretically, it is possible to create automatically models for analyzing larger and more complex data and to produce more accurate and repeatable results even on a large scale.

The application of these models would allow clinicians to identify new tasks, not merely diagnostic but also preventive, for major psychiatric disorders such as Schizophrenia.

## Conclusion

Approaches of big data, focusing on classification based on huge biological information rather than the single clinical manifestation, have the greatest advantage to move the field forward faster and with more evidence than before. The application of ML techniques in psychiatry as well, will be useful to routinely classify patients with major psychiatric disorders, and schizophrenia in particular, on the basis of resting state functional MRI data. This technique can be a valid, cheap, and non-invasive support for physicians to detect patients, even in the early stage of the disorder, conferring a crucial diagnostic anticipation, hopefully decisive in changing the natural history of the disease. The results collected in this review allow us to assume that the greater accuracy demonstrated by the SVM models and new integrated methods of ML techniques could play an increasingly decisive role in the future both for the early diagnosis and a more accurate evaluation of the treatment response, and to establish the middle-term prognosis of patients with SCZ.

## Author Contributions

All authors contributed to the article and approved the submitted version.

## Funding

This research was co-funded by University "G. Fortunato" Benevento (Italy). Grant: cda N°8/111119.

## Conflict of Interest

The authors declare that the research was conducted in the absence of any commercial or financial relationships that could be construed as a potential conflict of interest.
